# A STUDY OF CORRELATION BETWEEN TRANSHILAR DIAMETER AND P PULMONALE IN COPD PATIENTS

**DOI:** 10.4103/0970-2113.45278

**Published:** 2008

**Authors:** C Ravindran, KV Padmanabhan, Rejna Sreedhar

**Affiliations:** Department Pulmonary Medicine, Institute of Chest Diseases, Medical College, Calicut, India

**Keywords:** Transhilar Diameter, Hilar thoracic index, P pulmonale

## Abstract

**Background:**

Early diagnosis and therapy of Pulmonary hypertension in COPD patients may help in retardation of progression and amelioration of symptoms. This is an attempt to diagnose the disease from X-ray chest of COPD patients, so that invasive investigations can be avoided.

**Objectives:**

(1) Measurement of transhilar diameter in Chest X-ray PA view of COPD patients and its importance in detection of pulmonary hyper-tension. (2) To study correlation between P pulmonale and transhilar diameter / hilar thoracic ratio.

**Design:**

Prospective Clinical study.

**Setting:**

Institute of Chest Diseases, Medical College, Calicut.

**Period of study:**

One year from September 2002 to August 2003.

**Materials and Methods:**

100 patients admitted to Institute of Chest Diseases with COPD and ECG evidence of P pulmonale and/or RVH were included. Chest X-rays was taken and the following diameters were measured. Transhilar diameter, hilar thoracic ratio, width of descending branch of (Rt) pulmonary artery and cardiothoracic ratio.

**Conclusions:**

Chest X-ray can be used for identification of pulmonary hypertension in COPD patients. Positive correlation was seen between P pulmonale and hilar thoracic ratio. Positive correlation was also seen between P pulmonale and other parameters like smoking status, symptom duration, ECG evidence of RVH and negative correlation was seen with % predicted FEV_1_.

## INTRODUCTION

Pulmonary hypertension is a dreaded complication of COPD and has a very insidious onset. Patients usually remain asymptomatic until they manifest with features of overt RVH. Cor pulmonale is estimated to account for 6-7% of all types of adult heart disease in the united States, with chronic obstructive pulmonary disease (COPD) the causative factor in more than 50% of cases. COPD was the cause in 84% of the 100 cases of chronic cor pulmonale studied by Ben Jrad et al^1^, Although the prevalence of COPD in the United States is about 15 million, the exact prevalence of cor pulmonale is difficult to determine because it does not occur in all cases of COPD and the physical examination and routine tests are relatively insensitive for the detection of pulmonary hypertension. Early diagnosis and therapy may to an extent retard disease progression and ameliorate symptoms. Definitive diagnosis requires pulmonary artery catheterization which being an invasive and costly procedure, is not available for use in routine clinical practice. Chest X-ray and ECG being non-invasive and easily accessible diagnostic modalities, have an important role to play. This has much clinical significance in this era where newer revolutionary therapy for pulmonary hypertension has evolved. Many researchers have tried to draw attention to certain radiological findings in early cases of pulmonary hypertension and cor pulmonale. Comparing chest radiographic features with the pulmonary artery pressure obtained during cardiac catheterization in persons with COPD, Matthay et al^2^ observed that an increase in the diameter of the right descending PA to >16 mm on posteroanterior projection combined with an increase in the diameter of the left descending PA to >18 mm on left lateral projection correctly identified PAH in 45 of 46 patients.

Present study attempts to assess the suitability of using chest X-ray as a screening investigation and to study the correlation between radiological indices and P pulmonale in ECG in COPD patients.

## MATERIALS AND METHODS

100 patients admitted to Institute of Chest Diseases with signs and symptoms suggestive of COPD were included. Patient who showed evidence of fixed airflow obstruction with FEV_1_/ FVC < 70% and FEV_1_ < 80% with less than 15% reversibility on post bronchodilator testing and ECG evidence of P pulmonale and/or RVH^3^ were included. P pulmonale is diagnosed when the amplitude of P wave in Lead II is more than 2.5 mm. Exclusion criteria were pulmonary hypertension secondary to other causes and chest X-ray abnormalities like pleural effusion, collapse, consolidation and fibrosis. All the patients were subjected to chest X-ray and the following parameters were measured.

1. Transhilar diameter : Defined as sum of distances from midline to first radiographically visible upper lobe arterial divisions on either side.
2. Hilar thoracic ratio =
  $\frac{\mathrm{Transhilar}\;\mathrm{Diameter}}{\mathrm{Transverse}\;\mathrm{Thoracic}\;\mathrm{Diameter}}$
3. Width of descending branch of Rt.pulmonary artery Measured just below Rt.hilum at its widest point.
4. Cardiothoracic Ratio =
  $\frac{\mathrm{Caridac}\;\mathrm{Diameter}*}{\mathrm{Transverse}\;\mathrm{Thoracic}\;\mathrm{Diameter}}$

*Cardiac diameter – Sum of maximum horizontal distance from midline to Rt. & Lt. cardiac borders.

** Transverse thoracic diameter – Sum of maximal distances from midline to inner aspect of ribs on either side.

### Statistical Analysis:

1. Linear regression analysis: To assess relation between p wave amplitude and various radiological parameters, smoking status, symptom duration, RVH_1_ and % predicted FEV_1_.
2. Univariate analysis with cut off values of various indices were done.

## RESULTS

100 patients were included in the study. 98 were males and 2 were females. Main symptom duration was 10.4 years in male and 8 years in females. All patients were smokers with smoking scores ranging from 25-265 pack years. Moderate to severe air flow obstruction was seen as evidenced by Mean FEV_1_ of 30.86, FEV_1_ / FVC of 62.81.

**Table I:** Mean Radiological Indices

Transhilar Diameter	11.22cm
Hilar thoracic ratio	41.38%
Width of descending branch of Rt.pulmonary artery	22.75cm
Cardiothoracic ratio	45.75

Mean P wave amplitude: 3.3

**Table II:** Results of Univariate Analysis of Indices with proposed cut off Values for pulmonary hypertension

Measurement	Mean	SD	P value	Cut off value
Hilar-thoracic Index	41.38	3.19	<0.002*	>35
Width desc. Branch of Rt.PA	22.75	2.98	<0.01	>20
Transhilar diameter	11.22	0.43	<0.04	>11
Cardiothoracic ratio	45.75	3.95	<0.03	>43

Hilar thoracic Index – Most reliable parameter

**Table III:** Correlation of hilar thoracic index with various indices

Measurement	Correlation coefficient	Significance
Smoking status (pack years)	0.24	Positive correlation
Symptom duration (years)	0.15	Positive correlation
RVH in ECG	0.27	Positive correlation
% predicted FEV1	−0.131	Negative correlation

**Table IV:** Correlation of p wave amplitude with various indices

Measurement	Correlation coefficient	Significance
Transhilar diameter	0.49*	Positive correlation
Hilar thoracic index	0.31	Positive correlation
Width of descending branch of Rt.PA	0.289	Positive correlation
Cardiothoracic Ratio	0.21	Negative correlation

* Most significant correlation seen between Transhilar diameter and P wave amplitude.

## DISCUSSION

This study attempts to evaluate the use of chest X-ray as a screening modality for pulmonary hypertension in COPD patients. Kota G Chelty^4^ in an earlier study had analysed the chest X-rays of COPD patients with pulmonary hypertension and defined certain radiological indices as cut off for pulmonary hypertension - Hilarthoracic Index > 35%, Transhilar ratio >11cm, Descending branch of Rt.PA > 20mm, Cardiothoracic Ratio > 43. Our study is comparable to this study. A univariate analysis of our results with the proposed cut off values showed our values to be significantly above the proposed cut off values. Hilar thoracic index was the most reliable parameter in our study (Table II). In a study conducted by Lupi and co-workers^5^, hilar thoracic index was above 35 in 100 of the 100 patients with pulmonary hypertension but in only 3 of the 50 normal controls.

Mathay and co-workers^2^ found the width of descending branch of Rt.PA to be >16mm in 43 out of 46 patients with PAH but in only 2 of the 15 normal subject. Hicken and associates^6^ studied the transhilar diameters and found it to be above 10.5cm in patients with pulmonary hypertension. A weak correlation was seen between Hilar thoracic index and FEV_1_ in our study. Caesar A Kellar et al^7^ in his study showed a weak correlation between fall in FEV_1_ and development of pulmonary hypertension. In our study, a positive correlation was seen between Hilarthoracic Index and smoking status, symptoms duration and P wave amplitude (Table II). In patients with COPD, a high cardiothoracic ratio is highly sensitive and 100% specific for the presence of pulmonary hypertension. Although the chest radiograph may provide evidence for the presence of pulmonary hypertension, it cannot measure the degree of pulmonary artery pressure elevation.

Pulmonary hypertension leads to development of Right ventricular hypertrophy and Rt.atrial enlargement evidenced by P pulmonale and feature of RVH on ECG. Correlation studied showed a significant positive correlation between P wave amplitude and Hilar thoracic index. Positive correlation was also seen with Trans hilar diameter, cardiothoracic ratio and width of descending branch of Rt. Pulmonary artery (Table III).

Limitation of this study was that since accurate estimation of pulmonary artery pressures were not done correlation between severity of PAH and various radiological indices could not be done. We conclude that chest X-rays may be a useful screening investigation for pulmonary hypertension in COPD patients.

## CONCLUSION

Chest X-ray can be used for identification of pulmonary hypertension in COPD patients. Positive correlation was seen between P pulmonale and hilar thoracic ratio. Positive correlation was also seen between P pulmonale and other parameters like smoking status, symptom duration, ECG evidence of RVH and negative correlation seen with % predicted FEV_1_.
